# Spatial continuous modeling of early Cenozoic carbon cycle and climate

**DOI:** 10.1093/nsr/nwae061

**Published:** 2024-02-21

**Authors:** Yonggang Liu, Jian Zhang, Haoyue Zuo, Zhilin He, Jiaqi Guo, Qifan Lin, Haonan Yu, Jiawenjing Lan, Jing Han, Zhihong Song, Zihan Yin, Liang Zhao, Yongyun Hu, Zhengtang Guo

**Affiliations:** Laboratory for Climate and Ocean-Atmosphere Studies, Department of Atmospheric and Oceanic Sciences, School of Physics, Peking University, China; Laboratory for Climate and Ocean-Atmosphere Studies, Department of Atmospheric and Oceanic Sciences, School of Physics, Peking University, China; Laboratory for Climate and Ocean-Atmosphere Studies, Department of Atmospheric and Oceanic Sciences, School of Physics, Peking University, China; Key Laboratory of Cenozoic Geology and Environment, Institute of Geology and Geophysics, Chinese Academy of Sciences, China; Laboratory for Climate and Ocean-Atmosphere Studies, Department of Atmospheric and Oceanic Sciences, School of Physics, Peking University, China; Laboratory for Climate and Ocean-Atmosphere Studies, Department of Atmospheric and Oceanic Sciences, School of Physics, Peking University, China; Laboratory for Climate and Ocean-Atmosphere Studies, Department of Atmospheric and Oceanic Sciences, School of Physics, Peking University, China; Laboratory for Climate and Ocean-Atmosphere Studies, Department of Atmospheric and Oceanic Sciences, School of Physics, Peking University, China; Laboratory for Climate and Ocean-Atmosphere Studies, Department of Atmospheric and Oceanic Sciences, School of Physics, Peking University, China; Laboratory for Climate and Ocean-Atmosphere Studies, Department of Atmospheric and Oceanic Sciences, School of Physics, Peking University, China; Laboratory for Climate and Ocean-Atmosphere Studies, Department of Atmospheric and Oceanic Sciences, School of Physics, Peking University, China; State Key Laboratory of Lithospheric Evolution, Institute of Geology and Geophysics, Chinese Academy of Sciences, China; Laboratory for Climate and Ocean-Atmosphere Studies, Department of Atmospheric and Oceanic Sciences, School of Physics, Peking University, China; Key Laboratory of Cenozoic Geology and Environment, Institute of Geology and Geophysics, Chinese Academy of Sciences, China

## Abstract

A real spatial continuous modeling of climate and carbon cycle is developed, and tested for early Cenozoic from 60 Ma to 40 Ma.

The partial pressure of atmospheric CO_2_ (*p*CO_2_) is determined by the carbon cycle that involves climate, biogeochemistry, and plate tectonics. In this cycle, atmospheric CO_2_ is converted into organic carbon and carbonates by biological activities and silicate weathering, and buried as sediments. The sediments on the ocean floor are subducted into the Earth's interior, and subsequently converted back into CO_2_ and outgassed into the atmosphere. To fully understand the evolution of *p*CO_2_ and climate, a model that is able to simulate this entire cycle (termed here as Supercycle) with spatial structure is clearly needed. While the simulation of outgassing driven by plate tectonics is technically challenging and currently under development [1], the spatial continuous modeling of the climate as well as its interaction with silicate weathering is the most prohibitive task in terms of computational demand. In this work, we provide the first test of a real spatial continuous modeling of climate and silicate weathering (a simplified carbon cycle) on a geological timescale, as an exploration for the future simulation of a full Supercycle. Results also show that they produce significantly different results from those of currently available models.

For a long time, only zero-dimensional models (e.g. GEOCARB [2]) could be run due to the high computational demand of models that resolve the spatial structure. The first spatial model is GEOCLIM [3], but it relies on climate datasets pre-calculated using an atmospheric General Circulation Model (GCM) for a number of *p*CO_2_ under specific continental configurations. The climate at actual *p*CO_2_ (either simulated by the model or taken from proxies) is obtained through interpolation and the continental configuration is fixed during the time period of concern. The most recently developed model, the Spatial Continuous Integration (SCION) model [4], combined GEOCLIM with a zero-dimensional biogeochemical model, COPSE (Carbon-Oxygen-Phosphorus-Sulfur-Evolution) [5]. SCION is able to simulate the evolution of climate and *p*CO_2_ of the whole Phanerozoic based on the climate datasets that have been pre-calculated at key time slices which are ∼20 million years (Myr) apart [4]. The climate and silicate weathering in between the key time slices are again obtained by linear interpolation. Before performing the interpolation, however, global averages are calculated first at the relevant key time slices. Thus, the simulation by SCION is essentially zero dimensional for most of the time period, with spatial information only implicitly included.

Interpolation makes it easy for the SCION model to run for many hundreds of millions of years but inevitably induces errors, as exemplified in what is to follow. Climate may not change linearly with log_2_(*p*CO_2_) as normally assumed even for a fixed continental configuration. For example, the global mean surface air temperature (GMAT) of 40 Ma at 4 × CO_2_ (1 × CO_2_ is 280 ppmv) would be overestimated if it is obtained by interpolating from the values at 2 × CO_2_ and 7 × CO_2_ (Fig. 1a; more clearly seen in Fig. S1). The global total weathering flux would be similarly overestimated (Fig. 1b and Fig. S1). Moreover, if the GMAT and weathering flux at 55 Ma for 4 × CO_2_ are interpolated from values at 60 Ma and 40 Ma obtained for the same *p*CO_2_, their values would be underestimated, especially the GMAT (Fig. 1a). Therefore, a true spatial continuous model is needed when studying the evolution of the climate and silicate weathering for a time period in which they do not vary linearly with either log_2_(*p*CO_2_) or time.

**Figure 1.**
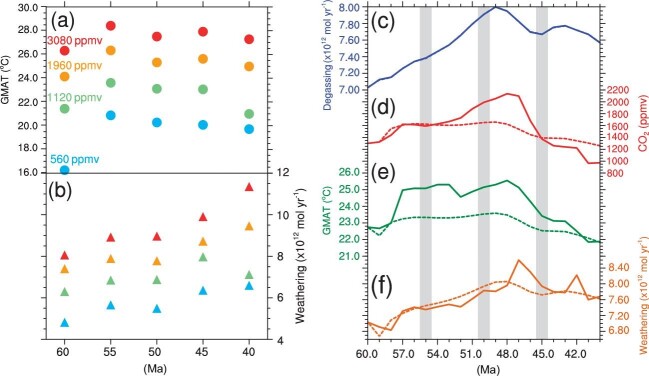
Simulation results of the climate and carbon cycle during the early Cenozoic. (a) Equilibrium GMAT (filled circles) simulated for five time slices between 60 Ma and 40 Ma at four prescribed *p*CO_2_: 2 × CO_2_ (blue), 4 × CO_2_ (green), 7 × CO_2_ (orange), and 11 × CO_2_ (pink). They demonstrate that the GMAT and weathering fluxes at 55 Ma, 50 Ma, and 40 Ma may not be accurately obtained by linear interpolation from the values at 60 Ma and 40 Ma as done in model SCION. (b) Global total weathering fluxes (triangles) calculated using the climate fields shown in (a). (c) The prescribed degassing rate for the transient simulation (see Supplementary Information for how this curve is constructed). (d–f) The evolution of *p*CO_2_, GMAT, and weathering flux simulated by coupled modeling of climate and carbon cycle (solid curves) and the simplified method of SCION (dashed curves).

Here we study the evolution of climate and *p*CO_2_ between 60 Ma and 40 Ma using an atmosphere-ocean GCM, CESM1.2, coupled to a silicate weathering model very similar to that of [6] which does not explicitly consider the influence of vegetation (see Supplementary Information for details). The weathering model used here differs from [6] only in the value of the erosion rate constant (*k_e_* in equation (8) of [6]; see Supplementary Information for explanation). Evolution during this time period was thought to be driven primarily by the subduction of the India plate [7], which induced an extra degassing of CO_2_ with its peak occurring at ∼49 Ma (Fig. 1c). The climate model and weathering model are coupled every 1 Myr. This time step is appropriate for the timescale of silicate weathering as a much shorter coupling interval would require the consideration of orbital variations. The degassing is not assumed to be balanced by silicate weathering, and the imbalance drives a change of *p*CO_2_ during each coupling interval. At the next coupling point, the climate is simulated for 200 years with this new *p*CO_2_ and the climate averaged over the last 30 years is used to calculate the weathering flux. Two hundred years are approximately enough for the simulated climate to reach equilibrium because the initial condition provided for each simulation is not far from that at equilibrium (Fig. S2). The whole process took ∼80 days to finish when run on 64 CPU cores.

The trend of *p*CO_2_ obtained with this model generally follows degassing, increasing during the first 11 Myr and decreasing afterwards (Fig. 1d). However, *p*CO_2_ at 40 Ma is smaller than that at 60 Ma despite a substantial increase of degassing rate during the whole period compared to that at 60 Ma (Fig. 1c). Correspondingly, the GMAT at 40 Ma is also lower than that at 60 Ma (Fig. 1e). Both the trend and values of the simulated *p*CO_2_ and GMAT are comparable to those of the reconstructions in [8]. The lower *p*CO_2_ at 40 Ma is clearly due to the evolution of the continent. For example, GMAT between 45 Ma and 40 Ma is much lower than those between 57 Ma and 52 Ma but the weathering flux is higher (Fig. 1f), driving a rapid decline of *p*CO_2_. This higher global weathering flux is due primarily to the higher tropical weathering flux which itself is mainly due to a larger continental area within the tropical region (Fig. S3).

If the SCION method were used, the variations of both GMAT and *p*CO_2_ would be quite mild and smooth during the early Cenozoic (dashed curves in Fig. 1d and e), while the global weathering flux would more closely follow that of the coupled model (Fig. 1f). However, a slight difference in weathering flux can induce a large difference in *p*CO_2_ over 10 Myr; the overestimation (up to 2.5%) during the first half of the early Cenozoic induces an underestimate of the peak *p*CO_2_ by 500 ppmv (1620 ppmv vs. 2120 ppmv). Moreover, the GMAT would be significantly underestimated over the whole period (Fig. 1d). This demonstrates the important influence of continental configuration at timescales much shorter than 20 Myr. When compared to reconstructions, the variations of both GMAT and *p*CO_2_ simulated by the model presented here are also superior to those simulated by SCION (Fig. S4).

A relatively small change in continental configuration may result in a large difference in GMAT (e.g. between 60 Ma and 55 Ma), especially when the climate is cold (filled circles in Fig. 1a), and a large difference in weathering flux (e.g. between 45 Ma and 40 Ma), especially when the climate is warm (triangles in Fig. 1b). The GMAT and weathering flux do not necessarily vary in the same direction, this is due to the different influence of continental configuration on them. Furthermore, the SCION method gives a different *p*CO_2_ at 40 Ma from that of the spatial continuous model (1250 ppmv vs. 970 ppmv), meaning that the errors within the 20 Myr period will accumulate and affect the evolution of climate and carbon cycle over a longer timescale.

The results above demonstrate not only the necessity of true spatial continuous modeling of the climate and carbon cycle in million-year timescales, but also the feasibility of running such models for a longer time period, e.g. the whole Cenozoic. With not too much addition of computational load, the biogeochemical cycle can be included in a relatively straightforward way since CESM1.2 used herein already has such an ability but was not turned on in this exploratory study. Therefore, it takes approximately one year of wall-clock time to run the fully coupled climate-carbon cycle model for the whole Cenozoic. More importantly, the present work demonstrates the possibility of long-term modeling of the Supercycle, which requires the sedimentation rate of both carbonate and organic carbon on the ocean floor as well as a geodynamic model that can explicitly simulate the degassing flux.

The present work also identifies the key problem that we need to pay attention to in the future. A few large jumps in GMAT (e.g. 57 Ma and 52 Ma; Fig. 1e) and weathering flux (e.g. 47 Ma and 42 Ma; Fig. 1f) appear in the simulation results. These are due to the sudden change of continental configuration at those time instants (Fig. S5), because paleogeographic reconstruction of the Cenozoic was performed for only once every ∼5 Myr [9]. Denser reconstruction of paleogeography is clearly needed, and may be obtained by combining the available reconstructions with the plate tectonic model [10].

## Supplementary Material

nwae061_Supplemental_File

## Data Availability

We are happy to share the model and data upon request.
