# Supplementary material for: Spatial continuous modeling of early Cenozoic carbon cycle and climate
Source: Natl Sci Rev. 2024 Feb 21;11(4):nwae061. doi: 10.1093/nsr/nwae061 (PMC10957155; doi:10.1093/nsr/nwae061)
Supplement: nwae061_Supplemental_File [file nwae061_supplemental_file.pdf]

Supplementary Information for "Spatial Continuous modeling of Early Cenozoic Carbon Cycle and Climate" by

Yonggang Liu, Jian Zhang, Haoyue Zuo, Zhilin He, Jiaqi Guo, Qifan Lin, Haonan Yu, Jiawenjing Lan, Jing Han, Zhihong Song, Zihan Yin, Liang Zhao, Yongyun Hu, Zhengtang Guo

The climate is simulated with the Community Earth System Model version 1.2.2 (CESM1.2.2), and the weathering is simulated by a model that is essentially the same as that in Park et al. [1]. The detailed description of the models, experimental setup, as well as the way the degassing flux is calculated are provided below.

Earth System Model

CESM1.2.2, which is developed and maintained by the National Center for Atmospheric Research (NCAR), includes modules of atmospheric, ocean, sea ice, land, and river routing. The atmospheric component, CAM4, is run at a relative coarse resolution of 3.75°×3.75° with 26 vertical levels. The land component, CLM4, has the same horizontal resolution with CAM4, but only 10 levels in the vertical. The ocean component, POP2, has 116 and 100 grid points in the meridional and zonal directions, respectively, and 60 levels in the vertical. To avoid singularity at the poles, the North Pole of ocean grid is placed on North America. The sea ice component, CICE4, shares the same horizontal resolution as POP2. The river routing module routes all runoff to the oceans with a resolution of 0.5°×0.5°. This version of CESM can yield a realistic simulation of the present climate [2], and has also been used to simulate deep paleo-climate [3, 4].

The ability of the dynamic vegetation module that comes with CESM1.2.2 in simulating the present-day vegetation distribution is quite unsatisfying [5, 6], so the model BIOME4 [7] is used instead. BIOME4 simulates the distribution of 28 biomes when atmospheric CO_2 concentration (pCO_2), monthly mean climate (precipitation,

temperature, shortwave radiation), and soil physical properties (water holding capacity and infiltration rate) are provided. This model simulates vegetation well and has been used in many paleoclimate simulations [7-11]. The BIOME4 and CESM1.2.2 exchange information once every 40 years. CESM1.2.2 is run for 40 years first, after which the monthly precipitation, temperature, and shortwave radiation averaged over the last 10 years are input into BIOME4. BIOME4 is run to equilibrium, and then the distribution of 28 biomes obtained are used to generate new land surface data for CESM1.2.2. This process is repeated until the simulation reaches quasi-equilibrium which takes approximately 500 years. The vegetation is pre-calculated for 60, 55, 50, 45, and 40 Ma whose paleogeography has been reconstructed (Fig. S5). $p\mathrm{CO_2}$ of 1820, 2800, 1960, 1540, and 1372 ppmv is used (the reason is given in the section Experimental design) when calculating the vegetation distribution for 60, 55, 50, 45, and 40 Ma, respectively.

Weathering model

The weathering model used here is basically the same as the model provided in Park et al. [1], but with the code written by ourselves in Python, and with some parameters retuned as described in what to follow. This weathering model is based on the framework of Gabet and Mudd [12], which explicitly considers the process of regolith generation and physical erosion in the vertical direction. The erosion rate depends on the surface slope and runoff. The surface slope is calculated from topography data provided in Scotese and Wright [13] using the first equation in the Appendix A of Maffre et al. [14]. The erosion rate constant in equation (8) of Park et al. [1], k_e, is often simplified to a globally uniform value and obtained by matching the total erosion fluxes 20Gt/yr estimated by Milliman and Farnsworth [15]. This value was 0.00291 in Park et al. [1] but is re-tuned to 0.0279 because we use different topography data [13] to calculate the surface slope. The reason we use topography data in Scotese and Wright [13] is because only low-resolution data are available for the deep time, we thought it would be better retune the parameter using low-resolution modern topography data. The kinetic constant of the chemical weathering depends on the surface temperature, runoff, and lithology, with the former two coming from

CESM1.2.2 simulations.

The present-day lithology distribution of Hartmann and Moosdorf [16] is used, which contains 16 lithological classes and we group them into 6 broader categories as done in Park et al. [1] (see Table S1 of their Supplementary Information). Since the paleo-lithology is unavailable, here we obtain them by employing the software GPlates [17] to retrogressively project the present-day lithology map back into its historical configuration. For missing values at certain places, linear interpolation has been used to fill in the gaps. Since the timescale concerned here is million years, the weathering of carbonates is not counted into the weathering flux. The lithology map obtained this way is certainly not ideal but is sufficient for the purpose of the current work.

Vegetation affects continental weathering both directly by changing erosion or exuding acid and indirectly by affecting climate. The latter is partially included because the vegetation was simulated for the five time slices 60, 55, 50, 45, and 40 Ma whose continental configurations were available. The direct effect of vegetation on silicate weathering is not considered because it is very uncertain and is not considered in the model of Park et al. [1].

Estimation of Degassing Rate

The variation of degassing rate drives the evolution of climate and $p\mathrm{CO_2}$ in our study, which itself is assumed to be driven by the variation of subducted slab flux in the India–Eurasia collision region from 60 Ma to present day [18]. Since there is no geodynamic model available yet that can reliably calculate the global degassing flux, this flux has to be estimated somehow. First, we assume that the global silicate weathering flux and degassing flux are equal at the beginning of the study period, that is, 60 Ma. The weathering flux can be calculated with the climate model and weathering model described above, which give a value of 7.05×10^{18} mol per Myr. This flux is used as the background global degassing flux. Then, we consider the degassing due to the India–Eurasia collision as an add-on to the background degassing.

The degassing is assumed to be proportional to the subduction flux of India plate (Fig. 4c of [18]). The proportion constant has to be determined such that pCO_2 can increase from ~1300 ppmv at 60 Ma to ~2000 ppmv at 50 Ma according to the reconstruction of [19]. This increase will require 1.27×10^{17} mole of CO_2 to be added to the atmosphere if pCO_2 is to increase from 1300 ppmv to 2000 ppmv, with the total number of moles of the atmosphere set to be 1.8211×10^{21} [20, 21]. However, because ocean and land may absorb most of CO_2 emitted from the solid Earth, the actual CO_2 that needs to be emitted must be much larger than 1.27×10^{17} mole. The fraction of CO_2 left in the atmosphere has been previously estimated to be 0.09 [22]. When this fraction is used, then 1.416×10^{18} mole of extra CO_2 needs to be emitted between 60 Ma and 50 Ma due to the subduction of India plate. Then a proportion constant of 1.008×10^{10} mole/km^3 is obtained between the degassing flux and the subduction flux. A time series of the degassing flux is obtained from that of the subduction flux accordingly. The time step of simulation is 1 Myr in the current study and the degassing flux is assumed to be varying linearly within each time step.

It is worth mentioning that on decadal time scales, studies showed that the ocean and land absorb approximately 50% of the excess atmospheric CO_2 in total emitted by human activities [23-25]. We use a low value here based on the consideration that the time scale of our study is million years, much longer than decadal time scale. At million year timescale, there will be enough time for CO_2 to enter the abyssal ocean rather than penetrating into only the upper ocean at decadal time scale [26]. Moreover, CO_2 that enters the abyssal ocean will react with both calcium carbonate and silicates on the seafloor, allowing the ocean to absorb more CO_2 [27].

Although we obtain the value 1.416×10^{18} mol above by considering the substantial absorption of CO_2 by oceans and land vegetation, these processes are not included in the current simulation. Therefore, it seems that we should have used the smaller value of 1.27×10^{17} mole. The much higher degassing flux is used here anyway based on the

strategy that we would like the final modelled peak CO_2 be overestimated rather than underestimated. It turns out that the final peak CO_2 obtained by the simulation is near 2000 ppmv (Fig. 1d of main text), probably indicating that the negative feedback due to silicate weathering is very efficient.

Experimental design

Before starting the simulations, the climate and vegetation are simulated using the coupled CESM1.2.2 and BIOME4 as described above for 60, 55, 50, 45, and 40 Ma. The pCO_2 for each time period is tunned so that the simulated global mean surface air temperature (GMAT) is the same as that reconstructed by Scotese et al. [28]. The final pCO_2 obtained are 1820, 2800, 1960, 1540 and 1372 ppmv for the five time periods, respectively. These simulations are continued for at least 2000 years and the GMAT is drifting at a rate smaller than 0.03 °C/100 yr in the final 300 years.

Two sets of experiments are performed in this study. The first set consists of 21 experiments, each of which represents a time-slice from 60 to 40 Ma with an interval of 1 Ma. The 21 experiments are run successively, and thus are time consuming. The climate simulation of 60 Ma is branched from the pre-calculated quasi-equilibrium state described above, but with pCO_2 set to 1300 ppmv and vegetation fixed. The model CESM1.2.2 is run for 200 years and the average of the final 50 years is used to force the weathering model. Once the weathering flux is obtained, pCO_2 for 59 Ma can be determined from the degassing flux. The 59 Ma experiment is branched from the end of 60 Ma experiment and again run for 200 years (Fig. S2). This process is repeated till 57 Ma, at which both the continental configuration and vegetation distribution are changed to that of 55 Ma (Fig. S5). The 57 Ma experiment is branched from the pre-calculated 55 Ma quasi-equilibrium state. The same processes of calculating climate, weathering, and pCO_2 above are then repeated for 5 Myr. The next nodal points at which the continental configuration and vegetation are changed are 52 Ma, 47 Ma, and 42 Ma. In the future, when continental configurations become available at every million year, the continental configuration and vegetation will be changed as frequently. In this set

of experiments, the solar constant is linearly increased from the 1354.48 to 1356.64 W/m^2 as the luminosity of Sun increases with time [29]. This set of experiments thus quasi-transiently simulate the evolution of climate and $p$$CO_2$ from 60 Ma to 40 Ma, and the solid curves in Fig. 1c-f of the main text are based on the results of these experiments.

The second set of experiments simulate the equilibrium climates of 60 Ma, 55 Ma, 50 Ma, 45 Ma, and 40 Ma at four different atmospheric $p$$CO_2$ levels, i.e. 560, 1120, 1960, 3080 ppmv. These four levels almost bracket the full range of $p$$CO_2$ estimated from geological reconstructions [19]. The total number of experiments in this set is thus 20. The vegetation distribution is fixed to that in their respective pre-calculated state. Every experiment is run for at least 2000 model-years. The GMAT and global weathering fluxes are also calculated for each experiment using the final 100 years of data. The data points in Fig. 1a, b of the main text are based on the results of this set of experiments (see also Fig. S3). The dashed curves in Fig. 1d-f are also calculated based on these results using the method of GEOCLM [30].

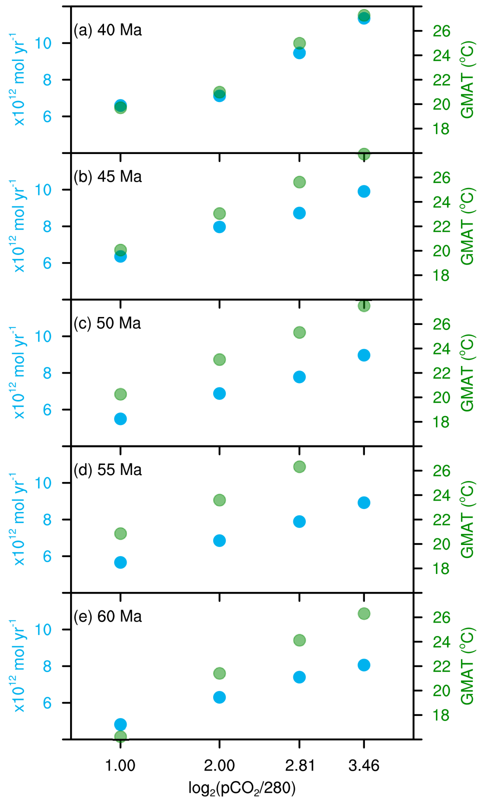

Figure S1 Equilibrium global mean surface air temperature (GMAT; green) and global total weathering fluxes (blue) simulated for five time slices between 60 Ma and 40 Ma at four prescribed pCO_2: 2×CO_2 (blue), 4×CO_2 (green), 7×CO_2 (orange), and 11×CO_2 (pink). They are the same as those in Fig. 1a of the main text but organized in a different way in order to demonstrate that GMAT and global total weathering fluxes may not vary linearly with log(pCO_2), most clearly seen in (a).

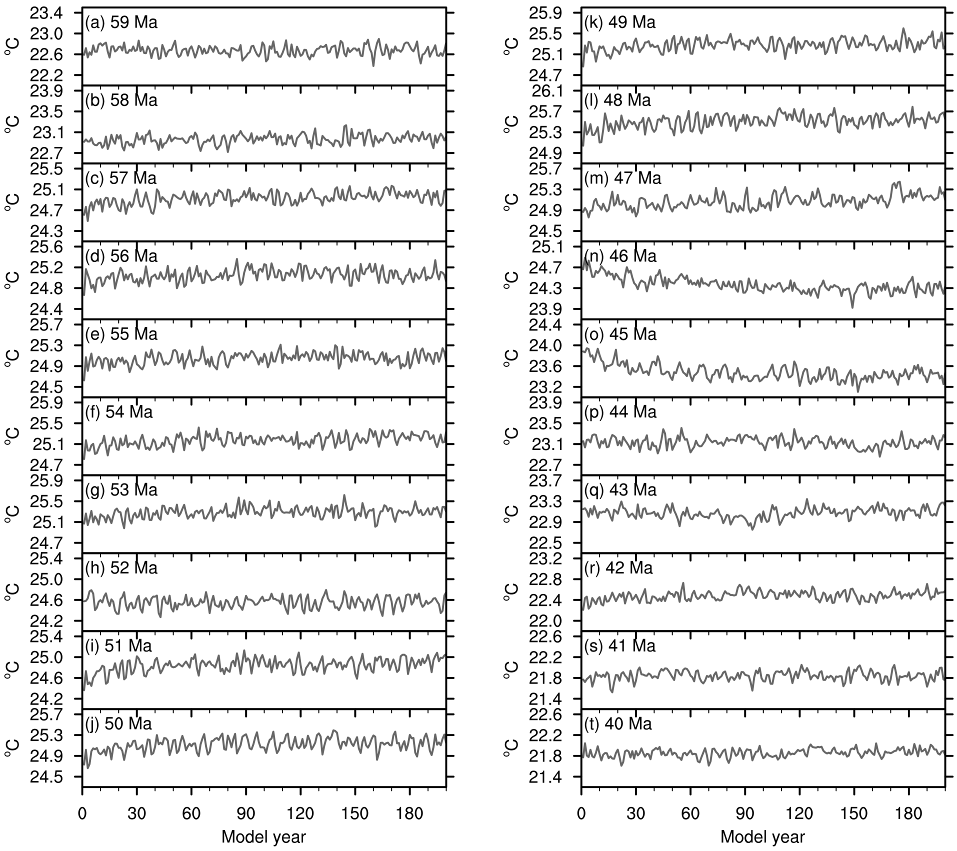

Figure S2 The time series of global mean surface air temperature for all 20 coupling points from 59 Ma to 40 Ma, with a timestep of 1 Myr.

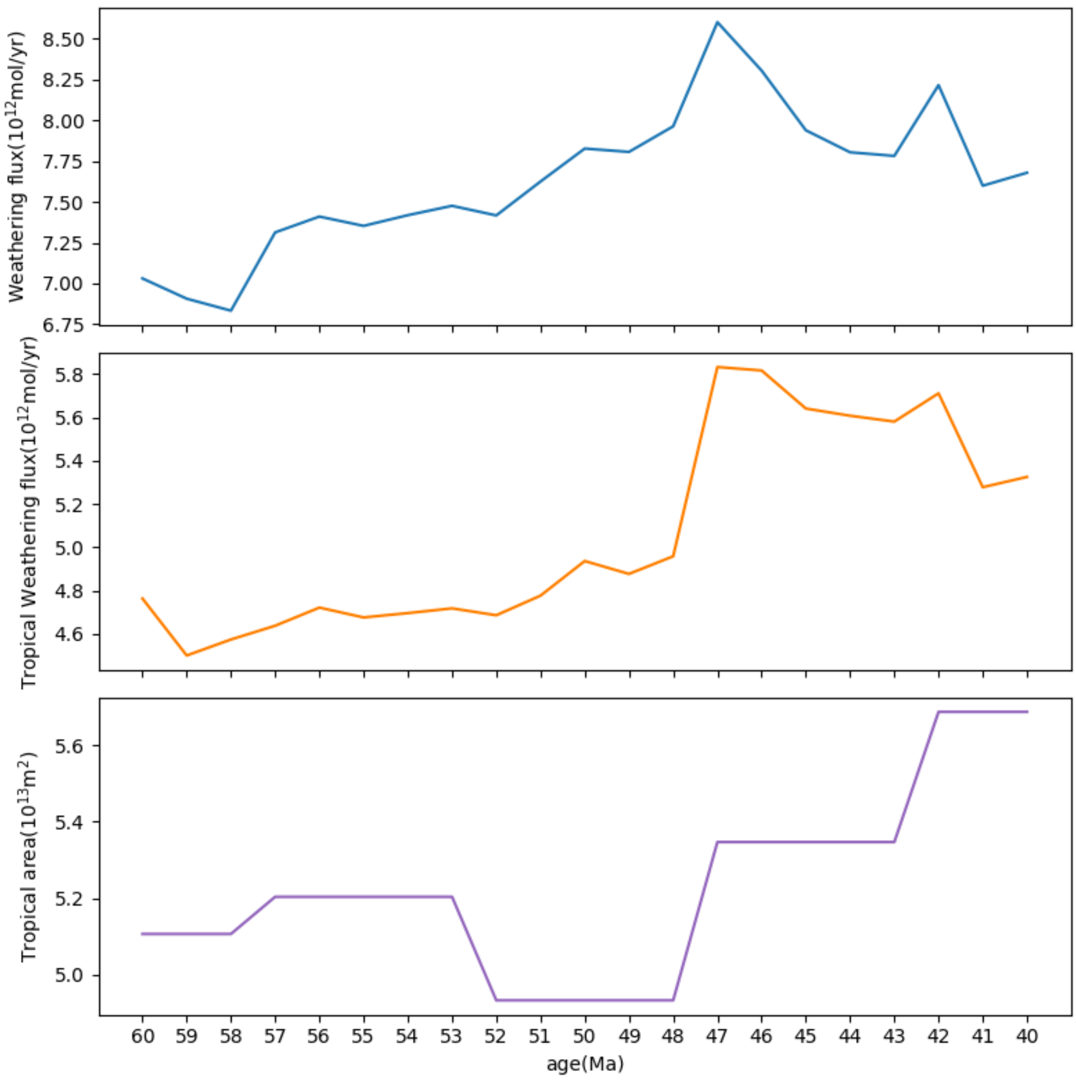

Figure S3 Time series of (a) global total weathering flux, (b) total weathering flux of the tropical region (30°S-30°N), and (c) the land area of tropical region (30°S-30°N).

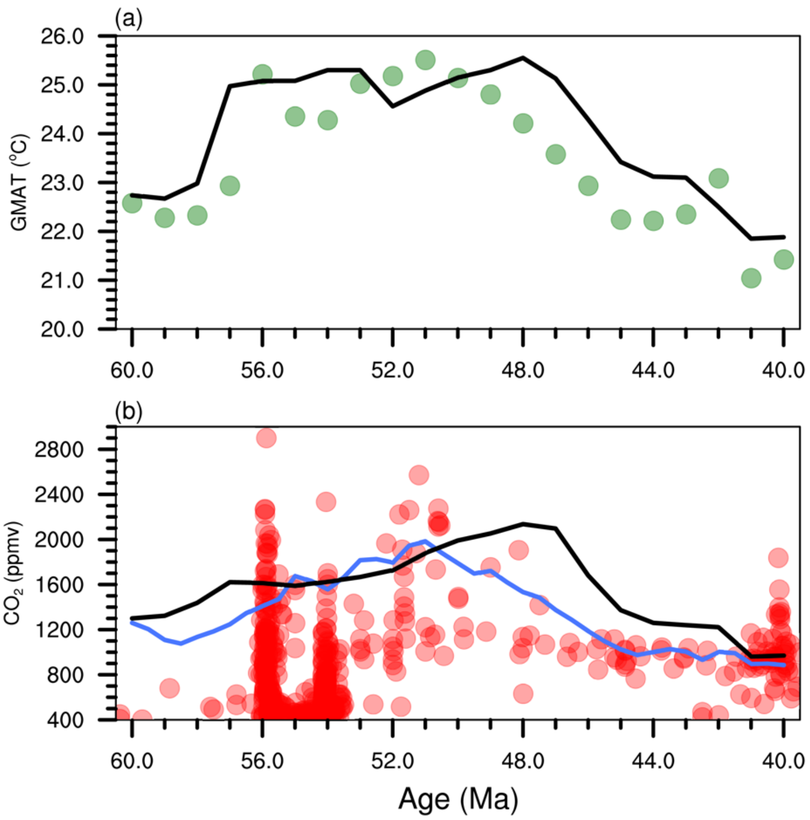

Figure S4 (a) Evolution of the modeled (black line) and reconstructed (green dots [28]) GMAT, and (b) pCO_2 from this study (black line), Hansen et al. [19], and proxy-reconstructions [31].

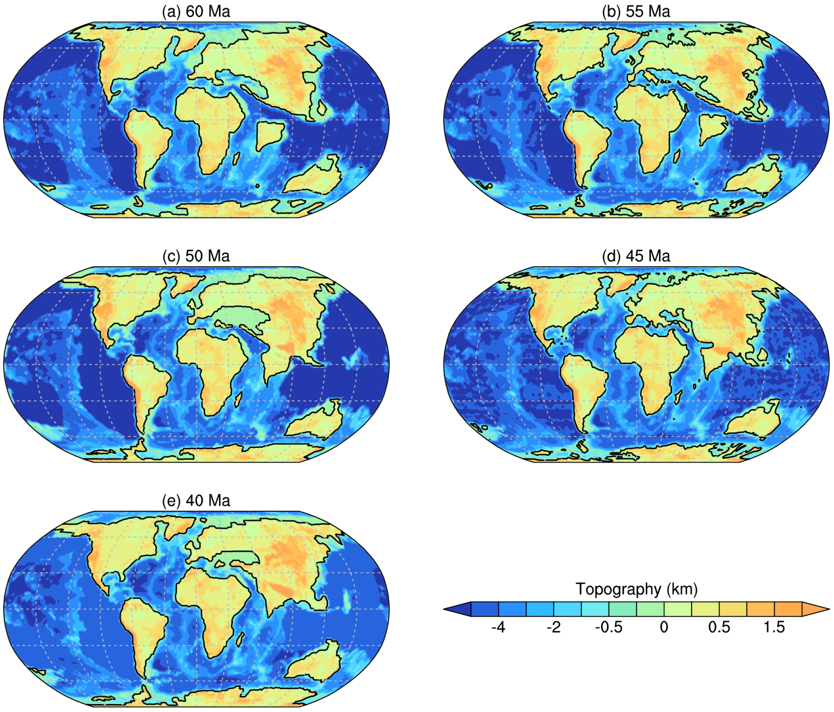

Figure S5 Continental distribution and surface topography for 60 Ma, 55 Ma, 50 Ma, 45 Ma, and 40 Ma from Scotese and Wright [13].

References

1. Park Y, Maffre P, Goddéris Y et al. *Proceedings of the National Academy of Sciences* 2020; **117**: 202011033.
2. Shields CA, Bailey DA, Danabasoglu G et al. *Journal of Climate* 2012; **25**: 3993-4014.
3. Fiorella RP and Sheldon ND. *Geology* 2017; **45**: 231-234.
4. Frieling J, Gebhardt H, Huber M et al. *Science Advances* 2017; **3**.
5. Li X, Hu Y, Guo J et al. *Scientific Data* 2022; **9**: 371.
6. Liu P, Liu Y, Peng Y et al. *Nature Communications* 2020; **11**: 4427.
7. Kaplan JO, Bigelow NH, Prentice IC et al. *Journal of Geophysical Research: Atmospheres* 2003; **108**.
8. Aciego SM, Heavens NG, Hinnov LA et al. *The Paleontological Society Papers* 2015; **21**: 83-120.
9. Albani S, Mahowald NM, Winckler G et al. *Clim. Past* 2015; **11**: 869-903.
10. Mahowald N, Kohfeld K, Hansson M et al. *Journal of Geophysical Research: Atmospheres* 1999; **104**: 15895-15916.
11. Albani S and Mahowald NM. *Journal of Climate* 2019; **32**: 7897-7913.
12. Gabet EJ and Mudd SM. *Geology* 2009; **37**: 151-154.
13. Scotese CR and Wright N. *Paleomap Proj* 2018.
14. Maffre P, Ladant J-B, Moquet J-S et al. *Earth and Planetary Science Letters* 2018; **493**: 174-185.
15. Milliman JD and Farnsworth KL, *River Discharge to the Coastal Ocean: A Global Synthesis*. 2011, Cambridge: Cambridge University Press.
16. Hartmann J and Moosdorf N. *Geochemistry, Geophysics, Geosystems* 2012; **13**.
17. Gurnis M, Turner M, Zahirovic S et al. *Computers & Geosciences* 2012; **38**: 35-42.
18. Shen H, Zhao L, Guo Z et al. *Science Bulletin* 2023; **68**: 637-644.
19. Hansen J, Sato M, Russell G et al. *Philosophical Transactions of the Royal Society A: Mathematical, Physical and Engineering Sciences* 2013; **371**.
20. Krissansen-Totton J and Catling DC. *Nat Commun* 2017; **8**: 15423.
21. Caves Rugenstein JK, Ibarra DE, and von Blanckenburg F. *Nature* 2019; **571**: 99-102.
22. Colbourn G, Ridgwell A, and Lenton TM. 2015; **29**: 583-596.
23. Watson AJ, Schuster U, Shutler JD et al. *Nature Communications* 2020; **11**: 4422.
24. Li C, Huang J, Ding L et al. *Earth's Future* 2021; **9**: e2021EF002124.
25. Piao S, Wang X, Park T et al. *Nature Reviews Earth & Environment* 2020; **1**: 14-27.
26. Jacob DJ. *Introduction to Atmospheric Chemistry*. 1999.
27. Penman DE, Caves Rugenstein JK, Ibarra DE et al. *Earth-Science Reviews* 2020; **209**: 103298.
28. Scotese CR, Song H, Mills BJW et al. *Earth-Science Reviews* 2021; **215**: 103503.
29. Gough DO. *Solar Physics* 1981; **74**: 21-34.
30. Arndt S, Regnier P, Goddéris Y et al. *Geosci. Model Dev.* 2011; **4**: 451-481.
31. Consortium TCCPIP, Hönisch B, Royer DL et al. *Science* 2023; **382**: eadi5177.
